# Cell death induction and intracellular vesicle formation in human colorectal cancer cells treated with Δ^9^-Tetrahydrocannabinol

**DOI:** 10.1007/s13258-023-01466-7

**Published:** 2023-10-14

**Authors:** Yu-Na Hwang, In-Seo Kwon, Ju-Hee Park, Han-Heom Na, Tae-Hyung Kwon, Jin-Sung Park, Keun-Cheol Kim

**Affiliations:** 1 Department of Biological Sciences, College of Natural Sciences, Kangwon National University, Chuncheon, Kangwon, 24341 Republic of Korea; 2 Kangwon Center for System Imaging, Chuncheon, Kangwon, 24341 Republic of Korea; 3 Chuncheon Bioindustry Foundation, Chuncheon, Kangwon, 24232 Republic of Korea; 4 Korean Pharmacopuncture Institute, Seoul, 07525 Republic of Korea

**Keywords:** Δ^9^-tetrahydrocannabinol (Δ^9^-THC), IC_50_, Cell death, Vesicle formation, PPARγ

## Abstract

**Background:**

Δ^9^-Tetrahydrocannabinol (Δ^9^-THC) is a principal psychoactive extract of Cannabis sativa and has been traditionally used as palliative medicine for neuropathic pain. Cannabidiol (CBD), an extract of hemp species, has recently attracted increased attention as a cancer treatment, but Δ^9^-THC is also requiring explored pharmacological application.

**Objective:**

This study evaluated the pharmacological effects of Δ^9^-THC in two human colorectal cancer cell lines. We investigated whether Δ^9^-THC treatment induces cell death in human colorectal cancer cells.

**Methods:**

We performed an MTT assay to determine the pharmacological concentration of Δ^9^-THC. Annxein V and Western blot analysis confirmed that Δ^9^-THC induced apoptosis in colorectal cancer cells. Metabolic activity was evaluated using MitoTracker staining and ATP determination. We investigated vesicle formation by Δ^9^-THC treatment using GW9662, known as a PPARγ inhibitor.

**Results:**

The MTT assay showed that treatment with 40 μM Δ^9^-THC and above inhibited the proliferation of colorectal cancer cells. Multiple intracytoplasmic vesicles were detected upon microscopic observation, and fluorescence-activated cell sorting analysis showed cell death via G1 arrest. Δ^9^-THC treatment increased the expression of cell death marker proteins, including p53, cleaved PARP-1, RIP1, and RIP3, suggesting that Δ^9^-THC induced the death of colorectal cancer cells. Δ^9^-THC treatment also reduced ATP production via changes in Bax and Bcl-2. Δ^9^-THC regulated intracytoplasmic vesicle formation by modulating the expression of PPARγ and clathrin, adding that antiproliferative activity of Δ^9^-THC was also affected.

**Conclusion:**

In conclusion, Δ^9^-THC regulated two functional mechanisms, intracellular vesicle formation and cell death. These findings can help to determine how cannabinoids can be used most effectively to improve the efficacy of cancer treatment.

**Supplementary Information:**

The online version contains supplementary material available at 10.1007/s13258-023-01466-7.

## Introduction

Phytocannabinoids, compounds from Cannabis sativa (Hemp species), contain Δ9-tetrahydrocannabinol (Δ^9^-THC), cannabidiol (CBD), cannabigerol (CBG), canna-bichromene (CBC), and other compounds (Bonini et al. 2018). Cannabinoid extracts exhibit pharmacological effects by regulating the endocannabinoid system (ECS) (Legare et al. 2022). Although some of the compounds has psychoactive activity, many concerns have been expressed regarding the effects of long-term therapeutic application (Sledzinski et al. 2020). CBD is a typical non-psychotropic com-ponent of hemp species (Koturbash and MacKay 2020). This compound is commonly used for chronic pain management in patients with advanced cancer, chronic pelvic pain, multiple sclerosis spasticity, fibromyalgia, and sleep apnea, as well as for adjunctive traditional analgesic therapy (Atalay et al. 2019; Britch et al. 2020; Hameed et al. 2023). The long-term administration of CBD led to clinically significant reductions in the frequency of convulsive and total seizures in patients with different epilepsy etiologies and also improved the quality of life of these patients (Silvestro et al. 2019). Animal experiments suggested that CBD diminished anxiety- and stress-related responses, suggesting that CBD might be effective for certain types of pain and emotional control (Batalla et al. 2020; Blessing et al. 2015; Svensson 2020). CBD is also known to have strong anti-cancer activity (Lee et al. 2022). Apoptosis and autophagy processes were involved in CBD-induced cytotoxicity of head and neck squamous cell carcinoma (Go et al. 2020). CBD exerts a highly cytotoxic effect, inducing cell death in triple-negative breast cancer MDA-MB-231 cells. However, IGF-1 and EGF antagonized the antiproliferative effect of CBD (D’Aloia et al. 2022). CBD treatment upregulated apoptosis-related proteins in A549 human lung cancer cells and induced vesicle-forming components, including PPARγ and clathrin, suggesting that CBD regulates both cell death and the differentiation of cancer cells (Park et al. 2022). Although terpenoids, another extract of hemp species, exert no therapeutic effect by themselves the synergistic effects of CBD and Δ^9^-THC in phytocannabinoids suggest that similar effects could be seen between terpene and phytocannabinoids (Russo 2011; Sommano et al. 2020).

Physiologically, the therapeutic activity of phytocannabinoids is mediated by the ECS comprised of CB1 and CB2 receptors (Kasatkina et al. 2021; Shalev et al. 2022). While CB1 is predominantly expressed in the central nervous system (CNS), CB2 is primarily expressed in various tissues such as spleen, thymus, and leukocytes (Howlett and Abood 2017; Liu et al. 2009; Marini et al. 2013). Both CB1 and CB2 receptors are activated by Δ^9^-THC and antagonized by CBD (Hua et al. 2017; Pertwee 2008). CBD exhibits high binding affinity to non-CB receptors, such as the G protein coupled receptor 55 (GPCR55), transient receptor potential vanilloid type 1 (TRPV1), and transient receptor potential cation channel subfamily M member 8 (TRPM8) (De Petrocellis et al. 2011). Although Δ^9^-THC shows many differences in pharmacokinetics and safety compared to CBD, it might be a promising compound with important pharmacological effects for disease treatment (Gherzi et al. 2020). However, the competitive antagonism of CB receptors might mean that Δ^9^-THC has a different action mechanism (Urits et al. 2020). Δ^9^-THC has been prescribed to cancer patients as a palliative medicine but not as an anticancer treatment (Hauser et al. 2017). Δ^9^-THC was recently reported to exert antiproliferative effects through the estrogen receptor (ER) in MCF-7 breast cancer cells and inhibit tumor angiogenesis (Schoeman et al. 2022). On the other hand, receptor targeted nanoparticles modulate anticancer activity through maximizing the amount of drug released in a sustained manner at the surface of cells, indicating that Δ^9^-THC-based antitumor therapies is highly promising approach (Duran-Lobato et al. 2022).

Phytocannabinoids have been explored as medical products for cancer treatment in many countries, and the effects of the numerous compounds have been studied (Lal et al. 2021). Therefore, it is necessary to evaluate the pharmacological effects of Δ^9^-THC as an anti-cancer drug compared to CBD. In this study, we analyzed the anticancer effect of Δ^9^-THC in colorectal cancer cells.

## Materials and methods

### Cell culture and reagents

We purchased the SW480 and LoVo human colorectal cancer cells from Korea Cell Line Bank (Seoul, Korea). The cells were maintained in DMEM medium (Welgene, Seoul, Korea) and RPMI-1640 medium (Welgene, Seoul, Korea). The media contained 10% fetal bovine serum and 1% penicillin and streptomycin. All cell lines were cultured at 37 °C in a 5% CO_2_ incubator (Thermo Fisher Scientific, MA, USA). We observed cellular morphology by phase contrast microscopy (Ts100, Nikon, Tokyo, Japan). Δ^9^-THC was purchased from the Cayman Company (Ann Arbor, MI, USA). Δ^9^-THC was dissolved in acetonitrile, and aliquots were stored at -20 °C.

### MTT assay

MTT (3-(4,5-dimethylthiazol-2-yl)-2,5-diphenyltetrazolium bromide) assay was performed to measure change in cell viability by Δ^9^-THC. 5X MTT solution (2.5 mg/mL) was prepared in phosphate-buffered saline (PBS). The cells were plated in 5x10^3^ cells for SW480 cells and 8x10^3^ cells for LoVo cells in each well, respectively. Next day, the cells were treated with different concentrations (0–80 μM) of Δ^9^-THC, and cultured for up to 96 h. Then, 200 μL of 1X MTT solution was added and incubated at 37 °C for 3 h. Formazan crystals were dissolved at room temperature for 30 min after replacing them with 100 μL of dimethyl sulfoxide (DMSO). Fluorescence was measured at excitation and emission wavelengths of 570 and 690 nm, respectively, using a microplate reader (Bio-Rad, Hercules, CA, USA). The experiment was performed as a triplicate. Fluorescence was measured at excitation and emission wavelengths of 570 and 690 nm, respectively, using a microplate reader (Bio-Rad, Hercules, CA, USA).

### Annexin V staining

The colorectal cancer cells were seeded in 6-well plates and cultured for 24 h at 37 °C and 5% CO_2_. The cells were treated with 40 μM Δ^9^-THC for 24 h. Cell death was analyzed using annexin V-FITC (BioVision, Milpitas, CA, USA). The cells were incubated with 50 μL of annexin V for 10 min in the dark at room temperature. The fluorescence positive cells were measured using a flow cytometer (FACSymphony, Bergen County, NJ, USA) and analyzed with FlowJo software (Tree Star, Ashland, OR, USA). Observable immuno-fluorescent cells were examined with a confocal microscope (Nikon, Minato, Tokyo, Japan).

### Flow cytometric analysis

The colorectal cancer cells were seeded in 60 mm dishes and cultured for 24 h at 37 °C and 5% CO_2_. Then, the cells were exposed to 40 μM Δ9-THC for 12, 24, 48, and 72 h. The cells were harvested, washed twice in PBS, and fixed in ice-cold 75% ethanol for 24 h. After fixation, the cells were stained with propidium iodide (50 μg/mL), and RNaseA (20 μg/mL) for 30 min. Cell cycle distribution was analyzed using flow cytometry (FACSymphony, Bergen County, NJ, USA) according to the manufacturer’s protocols.

### Western blot analysis

The colorectal cancer cells were seeded in 100 mm dishes and cultured for 24 h at 37 °C and 5% CO_2_. At the end of this culture period, the cells were exposed to 40 μM Δ^9^-THC for 12, 24, and 48 h. Proteins were extracted using RIPA lysis buffer (25 mM Tris-HCl pH 7.6, 150 mM NaCl, 1% NP-40, 1% sodium deoxycholate, 0.1% sodium do-decyl sulfate) containing proteasome inhibitors. Protein amounts were measured with a microplate reader using Bradford reagent (Thermo Fisher, Waltham, MA, USA). Samples with equal amount of proteins were subjected to SDS-PAGE and transferred to 0.45 μm polyvinylidene fluoride (PVDF) membrane. Next, we performed membrane blocking with 0.2% TBST solution with skim milk for 30 min at room temperature, and incubated with the primary antibody overnight at 4 °C. The membrane was washed with 0.2% TBST and incubated with secondary antibodies diluted in 5% skim milk at room temperature for 1 h. Finally, the proteins were detected on the film using ECL protein detection kit (GE HealthCare, Chicago, IL, USA). Primary antibodies for PARP-1, caspase 9, caspase 3, RIP1, and RIP3 were purchased from Cell Signaling Technology (Danvers, MA, USA). BAX, p53, and β-actin antibodies were purchased from Santa Cruz (Dallas, TX, USA). Clathrin, PPARγ, and β-adaptin antibodies were purchased from BD Biosciences (Franklin Lakes, NJ, USA), and Bcl-2 was obtained from Abcam (Cambridge, UK).

### qRT-PCR analysis

Total RNA was harvested and lysed with Trizol reagent (Ambion, North Greenbush, NJ, USA) and isolated by centrifugation with chloroform, followed by isopropyl alcohol, and lastly, by 75% ethanol. RNA was quantified using a Nano Spectrophotometer (BMG Labtech, Ortenberg, Germany). Complementary DNA (cDNA) was synthesized from 1 μg of total RNA from each extraction using random hexamer primers (Xenotech, Daejeon, Korea), following the manufacturer’s recommendations. Quantitative polymerase chain reaction (qPCR) analysis was performed with SYBR Green qPCR PreMIX (Enzynomics. Daejeon, Korea) according to the manufacturer’s instructions in a total volume of 20 μL, containing cDNA, qPCR mix, and primers. The PCR reaction conditions were: 95 °C for 10 min for initial denaturation, followed by 95 °C for 15 s and 60 °C for 1 min for 40 cycles. Melting curve analysis was performed from 60 to 95 °C with readings every 1 s, The 2^−ΔΔCT^ comparative method was used for the relative quantification of gene expression. The CT values were normalized to that of the β-actin housekeeping gene.

### Immunostaining

The colorectal cancer cells were cultured on 6-well plates with glass coverslips. After, the cells were treated with 40 μM Δ^9^-THC for 24 h, and then fixed with 4% paraformaldehyde solution for 20 min at 37 °C. The cells washed three times with PBS and permeabilized in PBS containing 0.1% Triton X-100. After that, the slides were blocked for 1 h in PBS with 5% skim milk, and followed by incubation with primary antibody (1:100) at room temperature for 1 h. Nuclei were counterstained with DAPI. After washing the cells with PBS twice, the cells were incubated with a secondary Alexa 488 goat anti-mouse IgG antibody (Abcam, Boston, MA, USA). The secondary antibody was diluted 1:200 in 5% skim milk and used. Finally, the slides were counterstained with DAPI. Fluorescent images were taken with the Kangwon Center for System Imaging’s confocal microscope (Ts2, Nikon, Tokyo, Japan).

### ATP determination

Cellular ATP levels were measured with an ATP Determination Kit reagent (Thermo Fisher, MA, USA). The standard reaction volume (10 mL) contains the following components: 0.5 mL 20X reaction buffer, 0.1 mL of 100 mM dithiothreitol, 0.5 mL of 10 mM D-luciferin, and 2.5 μL of 5 mg/mL firefly recombinant. Δ^9^-THC-treated cell lysate was prepared using a 1X luciferase lysis buffer. Afterward, the cell lysate was analyzed for bioluminescence activity using a luminometer (BioTek, Winooski, VT, USA).

### MitoTracker

Fluorescence-based MitoTracker was utilized to investigate the disruption of mitochondrial function according to manufacturer’s protocol (Thermo Fisher, Waltham, Massachusetts, USA). Briefly, the colorectal cancer cells were incubated on the coverslips of the culture dishes. Next day, the cells were treated with 40 μM Δ^9^-THC for 24 h. The cells on a coverslip were incubated with MitoTracker for 30 min at 37 °C. The coverslips were mounted on a microscope slide with Immu-Mount solution to prevent discoloration (Thermo Fisher, Waltham, MA, USA). The fluorescence images were taken with the Kangwon Center for System Imaging’s confocal microscope (Ts2, Nikon, Tokyo, Japan).

### Statistical analysis

GraphPad Prism 8.0 and ImageJ were utilized for statistical analyses. Graphs show the mean ± standard deviation (SD) from at least three independent experiments. Statistical significance was determined using the student’s t-test, with p-values ≤ 0.05 indicating statistical significance.

## Results

### Δ^9^-THC treatment shows cell death and morphological changes in human colorectal cancer cells

**Fig. 1:**
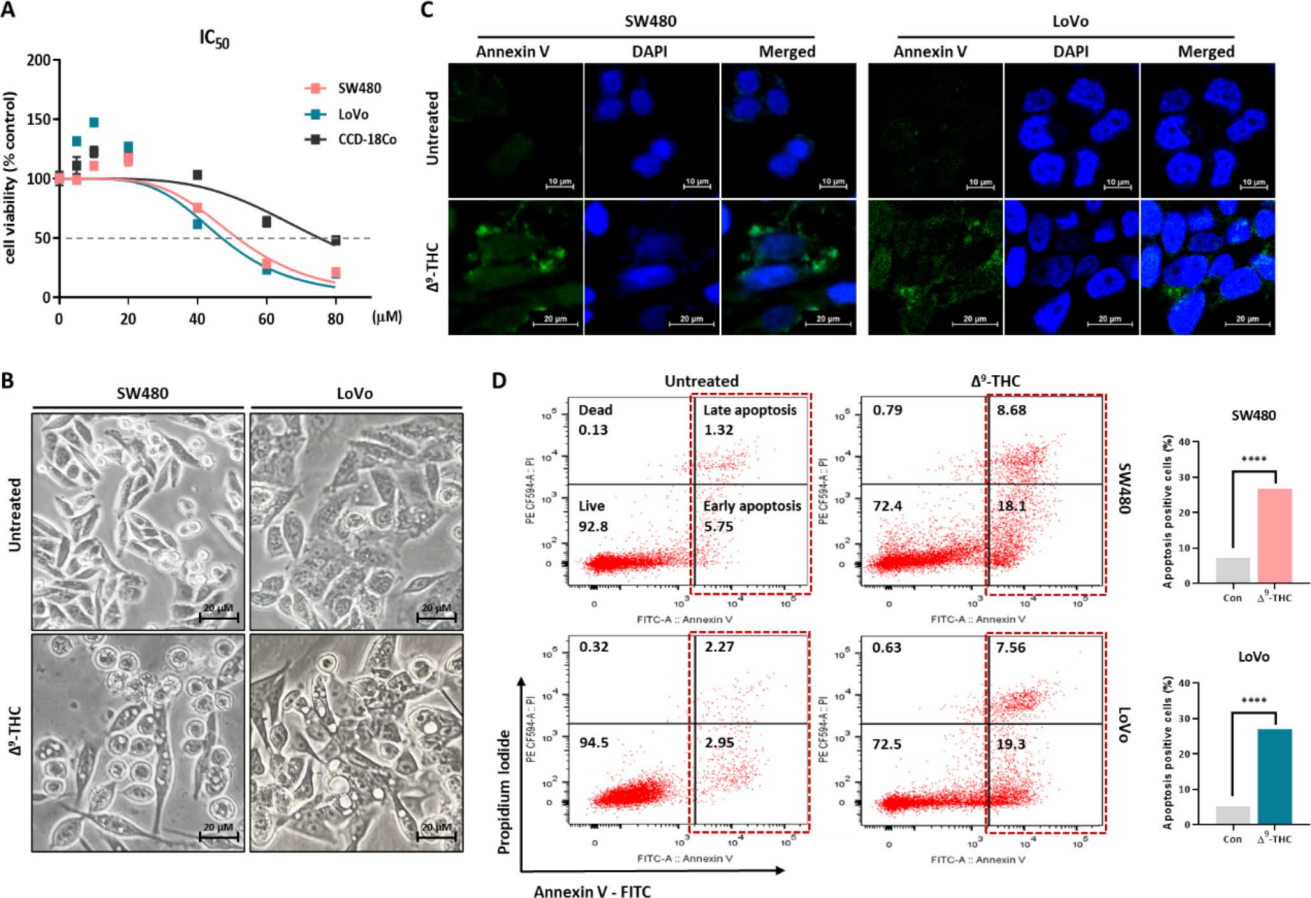
Growth inhibition and cell death effects by Δ^9^-THC treatment, according to concentration and treatment time. (**A**) The MTT assay was performed to confirm growth inhibition in SW480 and LoVo colorectal cancer cells by Δ^9^-THC treatment. Growth inhibition by Δ^9^-THC treatment was seen in the two colorectal cancer cell lines in dose- and time-dependent manners. The IC_50_ value was calculated by GraphPad Prism (CCD-18co = 75.15, SW480 = 51.48, and LoVo = 46.97 μM). (**B**) Cell morphology was dramatically changed by Δ^9^-THC treatment for 24 h. Numerous vesicles were observed in the cytoplasmic region. (**C**) Annexin V-FITC fluorescence staining analysis was performed in human colorectal cancer cells treated with Δ^9^-THC for 24 h. (**D**) left: Annexin V/FITC/PI flow cytometry analysis was performed to quantify cell death in human colorectal cancer cells treated with Δ^9^-THC for 24 h. right: Graph showed percentage of apoptotic cells in SW480 and LoVo cells

We performed an MTT assay to analyze the effects of Δ^9^-THC treatment on the proliferation of normal colon cells (CCD-18co) and human colorectal cancer cells (SW480 and LoVo) (Fig. 1A). We did not detect growth inhibitory effect at the concentration of 40 μM Δ^9^-THC in normal CCD-18co cells. No growth inhibition was observed by treatment with less than 20 μM Δ^9^-THC. However, distinct growth inhibition was observed at concentrations over 40 μM Δ^9^-THC in SW480 and LoVo colorectal cancer cells. The IC_50_ values of the SW480 cells and LoVo cells were 51.48 μM and 46.97 μM, respectively. We also observed intracellular vesicle formation in colorectal cancer cells treated with Δ^9^-THC (Fig. 1B). Vesicle formation was more prominent in LoVo cells than in SW480 cells, suggesting that this was due to differences in the genetic background of the two colorectal cancer cells. Δ^9^-THC treatment showed observable annexin-V-positive cells, indicating that this concentration of Δ^9^-THC could induce apoptotic cell death. (Fig. 1C). Quantitatively, the annexin V/PI flow cytometry experiment showed a 7.07–26.78% increase in fluorescence-positive SW480 cells treated with Δ^9^-THC and 5.22–26.86% in LoVo cells (Fig. 1D). Therefore, these results suggest that Δ^9^-THC treatment induced cell death by inhibiting cell growth and inducing cellular morphological changes.

### FACS analysis and western blot analysis show dual aspects for cell death and change in cell cycle distribution

**Fig. 2:**
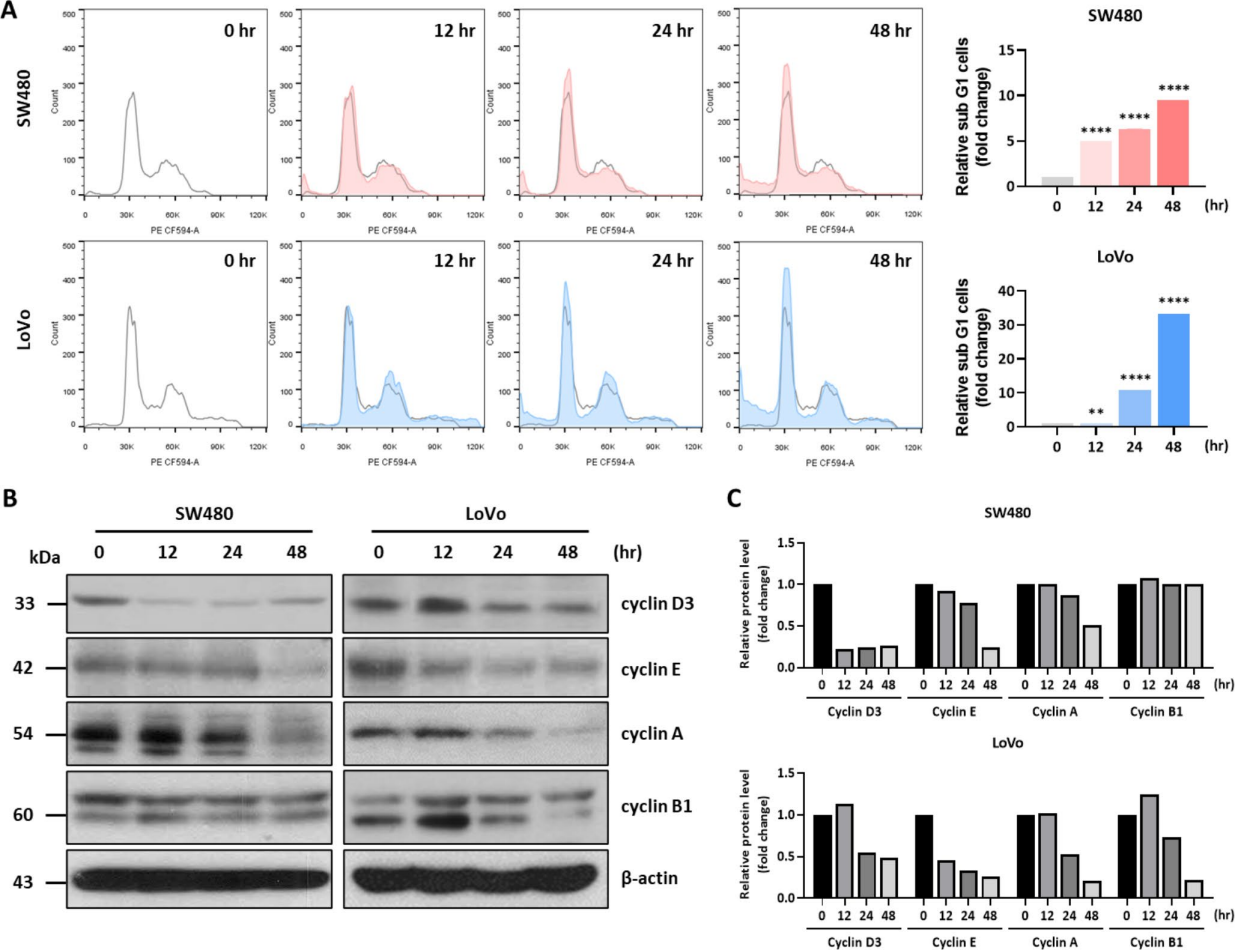
Cell cycle changes by Δ^9^-THC treatment analyzed by FACS. (**A**) left: Flow cytometric analysis performed following treatment of SW480 and LoVo human colorectal cancer cells with 40 μM Δ^9^-THC for 12, 24 and 48 h. G1 arrest occurred from 24 h, and cell death along with G1 arrest was observed from 48 h. right: Quantitative analysis graph of cell cycle distribution. (**B**) Changes in the expression of cell cycle-related proteins were confirmed by Western blot analysis. Cyclin E and A expression decreased when SW480 and LoVo cells were treated with Δ9-THC for 12, 24 and 48 h. No significant change in cyclin B1 was observed. (**C**) Densitometric analysis was performed with Western blot images.

FACS analysis was performed to examine cell cycle distribution after Δ^9^-THC treatment. There were no distinct cell cycle changes in colorectal cancer cells after 12 h of treatment with Δ^9^-THC. However, Δ^9^-THC treatment showed mild G1 arrest after 24 h and a number of dead cells after that (Fig. 2A). Interestingly, the majority of colorectal cancer cells also showed cell cycle distribution despite Δ^9^-THC treatment, suggesting that the inhibition of cell growth might have been due to cell morphological changes, including vesicle formation. We also performed the Western blot analysis of cell cycle-related proteins to confirm cell cycle alteration by Δ^9^-THC treatment. There were no distinct changes in cyclin proteins in treatment condition of vehicle (acetonitrile) (Supplementary Figure S1). However, most of the cyclins analyzed, including cyclin D3, cyclin E, and cyclin A, were decreased by Δ^9^-THC treatment, indicating that Δ^9^-THC treatment disrupted cell cycle-regulating proteins (Fig. 2B). Densitometric analysis of Western blot images obtained from Δ9-THC-treated cell lysates was performed (Fig. 2C). Most of the cyclin proteins were decreased by Δ^9^-THC treatment in a time-dependent manner. The decreased expression of cyclin proteins in response to Δ^9^-THC treatment might be a process for exiting from the normal cell cycle distribution. Therefore, Δ^9^-THC treatment not only induced death in human colorectal cancer cells but also changed the cell cycle distribution.

### Δ^9^-THC regulates proteins involved in cell death mechanisms

**Fig. 3:**
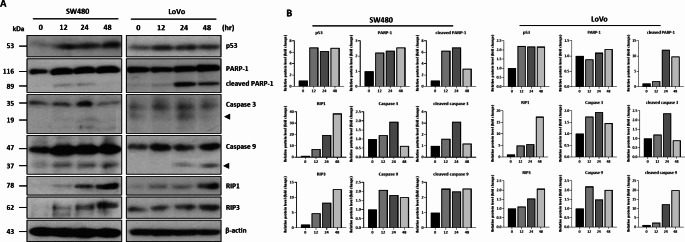
Western blot analysis of cell death-related proteins in Δ^9^-THC-treated colorectal cancer cells. (**A**) Western blot analysis was performed on SW480 and LoVo cancer cells treated with 40 μM Δ^9^-THC for 12, 24 and 48 h. The expression of p53, RIP1, and RIP3 were increased in Δ^9^-THC-treated cells, as well as cleaved forms of PARP, caspase 3, and caspase 9 were also observed. (**B**) Densitometric analysis was performed with Western blot images obtained from Δ^9^-THC treated SW480 and LoVo cell lysates

To examine the mechanism of cell death induction by Δ^9^-THC, we examined the expression of cell death-related proteins by Western blot analysis (Fig. 3A). Δ^9^-THC treatment increased the expression of p53 tumor suppressor protein and also the cleaved forms of caspase 3, caspase 9, and PARP-1, which are typical programmed cell death marker proteins. We also examined the expression of RIP1 and RIP3, which are markers for necroptotic cell death. Δ^9^-THC treatment increased the expression of RIP1 and RIP3 in both colorectal cancer cell lines. Densitometric analysis also suggested that the death related proteins were quantitatively changed in the Δ^9^-THC treated colorectal cancer cells (Fig. 3B). These results suggest that Δ^9^-THC regulates multiple cell death mechanisms, including apoptosis and necroptosis.

### Δ^9^-THC treatment changes ATP production by altering level of BAX and BCL-2

**Fig. 4:**
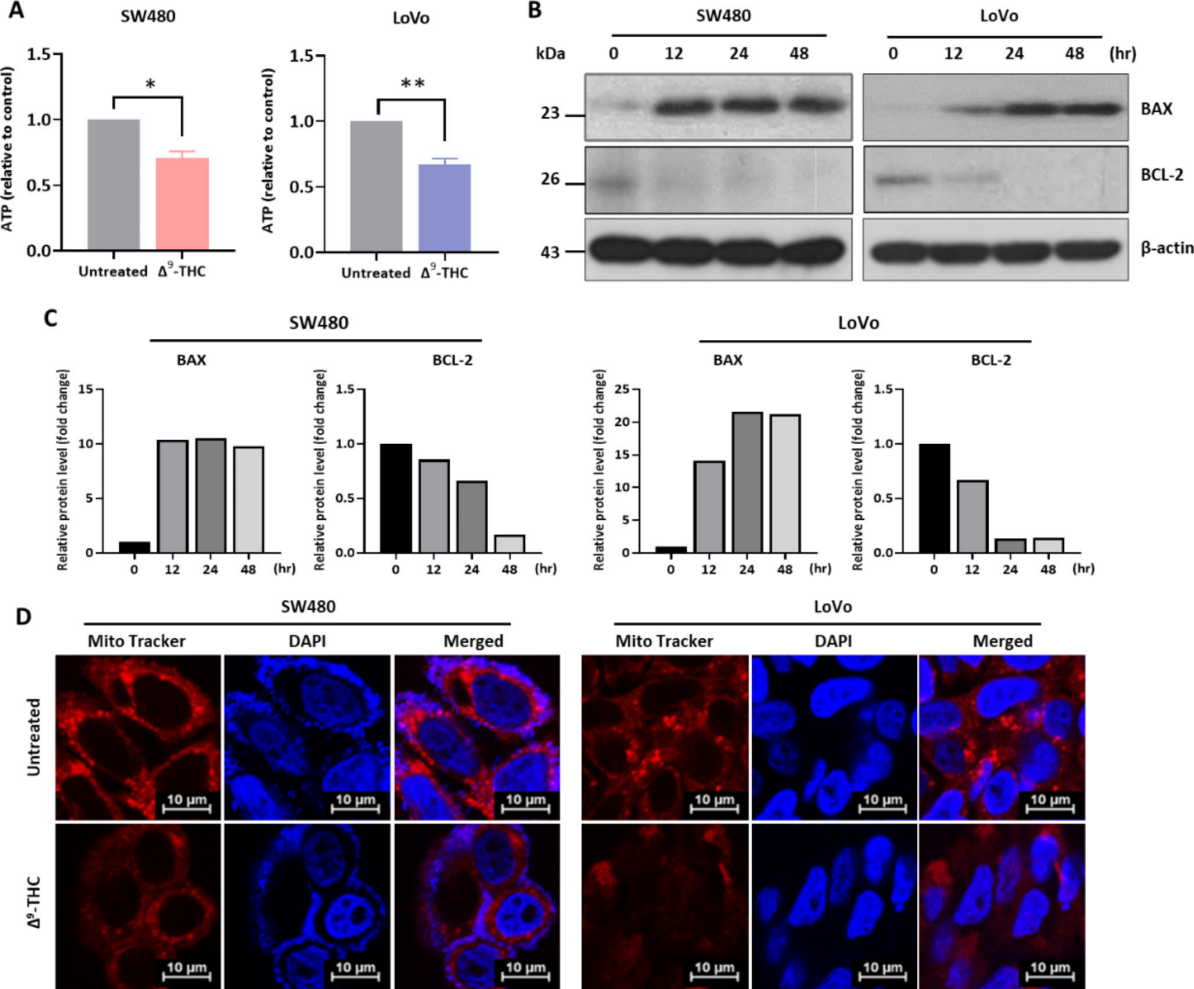
Regulation of mitochondrial function by Δ^9^-THC. (**A**) Intracellular ATP levels were determined after treatment with 40 μM Δ^9^-THC for 48 h. (**B**) Western blot analysis of SW480 and LoVo cells treated with 40 μM Δ^9^-THC for 12, 24 and 48 h. BAX expression increased with Δ^9^-THC treatment, whereas BCL-2 expression decreased. (**C**) Densitometric analysis was performed with Western blot images obtained from Δ^9^-THC treated SW480 and LoVo cell lysates. (**D**) SW480 and LoVo cells were treated with 40 μM Δ^9^-THC for 24 h and subjected to MitoTracker staining

We investigated whether changes in cellular characteristics were associated with changes in metabolic activity. To investigate the changes in metabolic activity, we analyzed ATP production in Δ^9^-THC treated colorectal cancer cells (Fig. 4A). Δ^9^-THC treatment slightly decreased intracellular ATP levels, suggesting that Δ^9^-THC altered cellular metabolic activity. Western blot analysis showed that Δ^9^-THC treatment also upregulated BAX and downregulated BCL-2, indicating that ATP production might be associated with changes in mitochondrial membrane proteins (Fig. 4B). Densitometric analysis also suggested that BAX and BCL-2 were quantitatively changed in the Δ^9^-THC treated colorectal cancer cells (Fig. 4C). MitoTracker is used for mitochondrial staining to study the cell cycle or processes such as apoptosis (Mauro-Lizcano et al. 2015). MitoTracker staining analysis showed heavily stained mitochondria in Δ^9^-THC-untreated cells, but the number of stained mitochondria was reduced upon Δ^9^-THC treatment (Fig. 4C). These results suggest that cell death and cytoplasmic vesicle formation by Δ^9^-THC treatment might be associated with changes in metabolic activity via changes in BAX and BCL-2.

### Δ^9^-THC regulates vesicle-forming proteins at the transcriptional level

**Fig. 5:**
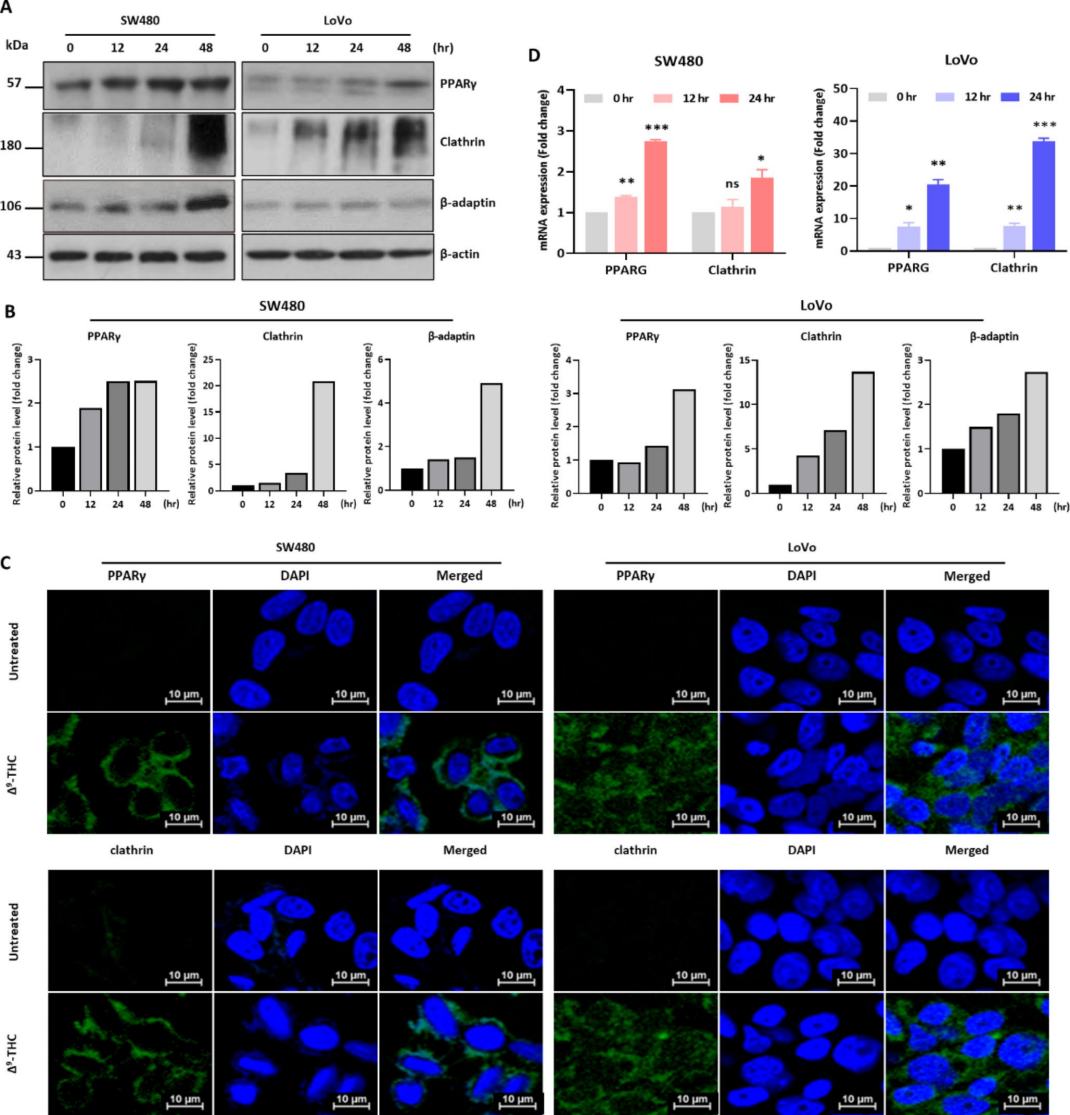
Regulation of vesicle formation by Δ^9^-THC. (**A**) Western blot analysis was performed in SW480 and LoVo cells treated with 40 μM Δ^9^-THC for 12, 24 and 48 h. Changes in the expression of vesicle formation-related proteins were confirmed by Western blot analysis. (**B**) Densitometric analysis was performed with Western blot images obtained from Δ^9^-THC treated SW480 and LoVo cell lysates. (**C**) PPARγ and clathrin expression was analyzed by immunostaining. Immunostaining was performed in SW480 and LoVo cells treated with 40 μM Δ^9^-THC for 24 h. (**D**) qRT-PCR analysis was performed in SW480 and LoVo cells treated with 40 μM Δ^9^-THC for 12 and 24 h. The expression of vesicle formation-related genes was changed by Δ^9^-THC treatment

The expression of PPARγ and clathrin, which are involved in cell morphological changes and vesicle formation, were investigated. Western blot analysis showed that the expression of PPARγ, clathrin, and β-adaptin was upregulated in both cancer cells by Δ^9^-THC treatment (Fig. 5A). Densitometric analysis also suggested that vesicle forming proteins were quantitatively changed in the Δ^9^-THC treated colorectal cancer cells (Fig. 5B). Immunostaining experiments showed PPARγ and clathrin in the cytoplasmic region (Fig. 5C). We also performed qRT-PCR analysis of PPARγ and clathrin gene expression using primer sets for these genes (Supplementary Table S1). These genes were increased by Δ^9^-THC treatment in a time-dependent manner (Fig. 5D). Therefore, Δ^9^-THC treatment regulated vesicle-forming proteins in these cells at the transcription level.

### PPARγ and vesicle formation are functionally interconnected

**Fig. 6:**
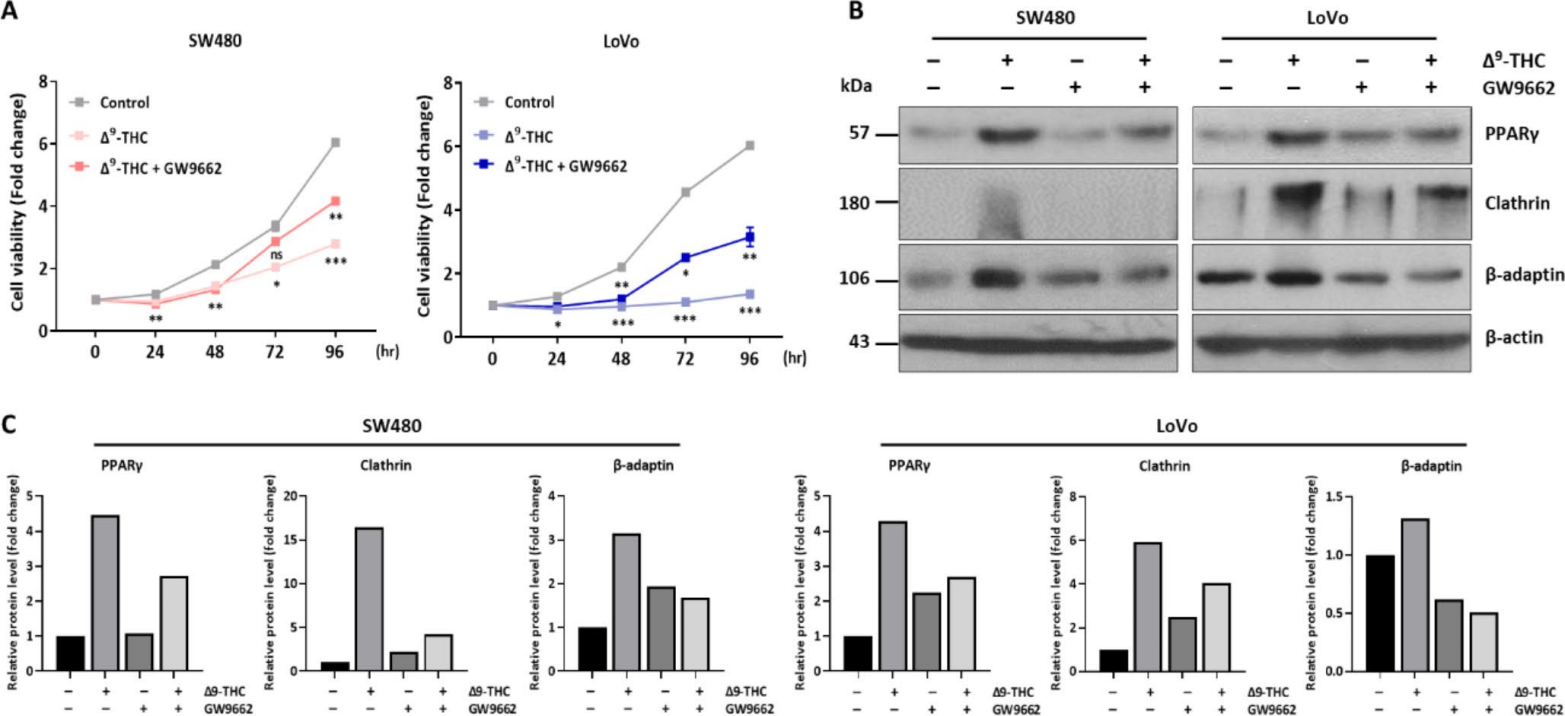
Regulation of vesicle formation by PPARγ. (**A**) The MTT assay was performed after combined treatment with Δ^9^-THC and GW9662, a PPARγ inhibitor. (**B**) Western blot analysis was performed in SW480 and LoVo human colorectal cancer cells treated with 40 μM Δ^9^-THC for 24 h, 3.3 nM GW9662, or the combination. (**C**) Densitometric analysis was performed with Western blot images

The mechanism of cell death and vesicle formation by Δ^9^-THC is very similar to that of CBD treatment in human cancer cells (Park et al. 2022). To investigate PPARγ relevance with Δ^9^-THC, we used GW9662, a PPARγ inhibitor, and Δ^9^-THC to analyze the molecular networks involved in vesicle formation changes. Generally, cell growth was less inhibited by combined Δ^9^-THC and GW9662 treatment (Fig. 6A). This growth inhibition was shown after 48 h cotreatment of Δ^9^-THC and GW9662. Western blot analysis consistently showed that the increased expression of vesicle formation-related proteins, such as clathrin and β-adaptin, was decreased by coadministration with GW9662 (Fig. 6B). Densitometric analysis also suggested that vesicle forming proteins were quantitatively changed in the Δ^9^-THC and GW9662 treated cells (Fig. 6C). Similarly, an immunostaining experiment showed that clathrin was increased by Δ^9^-THC treatment in the cytoplasmic region, but this was prevented by cotreatment with GW9662 (Supplementary Figure S2). Therefore, we suggest that vesicle formation might be closely associated with PPARγ activity during Δ^9^-THC treatment.

## Discussion

Phytocannabinoids extracted from Cannabis sativa have been examined for pharmacological applications in many disease models (Casey et al. 2022; Giuliano et al. 2021). Most cannabinoid extracts showed no significant cell cytotoxicity in normal fibroblasts (Milian et al. 2022). No changes in the viability of non-tumorigenic bladder cells (HBIEpC), epithelial cells (HMEC), or monocytes (TIGK) from Δ^9^-THC treatment were reported, indicating that Δ^9^-THC could have special advantages in treating various types of cancers (Caffarel et al. 2006; Gu et al. 2019; Whynot et al. 2023). Consistently, our current study indicated that Δ^9^-THC showed no distinct growth inhibitory effect in normal colon cells compared to colorectal cancer cells. While many studies on CBD have suggested it as a promising drug for cancer treatment, little is known about the pharmacological activity of Δ^9^-THC (Huang et al. 2021; Zagzoog et al. 2020). Δ^9^-THC has shown pharmacological activity in chronic pain and neurological diseases (Pertwee 2008). Δ^9^-THC reduced the Aβ load in 5XFAD/APP mice and restored cerebral glucose metabolism in the hippocampus of in vivo mouse models of sporadic Alzheimer’s disease (Chen et al. 2013). Δ^9^-THC was observed to have a direct neuroprotective effect in Parkinson’s disease-induced H-SY5Y cell cultures (Carroll et al. 2012). This neuroprotective effect was correlated with the increased expression of CB1R in a marmoset Parkinson’s disease model (Cooray et al. 2020). Δ^9^-THC has been suggested to have anticancer effects similar to those of CBD in many cancer cells. However, the pharmacological effects of Δ^9^-THC have not yet been fully explored at the molecular level (Semlali et al. 2021; Yang et al. 2020).

In this study, we investigated the anticancer effects of Δ^9^-THC in colorectal cancer cells. The human colorectal cancer cells used in this study, SW480 and LoVo cells, have slightly different genetic backgrounds. The oncogenic expression is similar, but SW480 cells are known to be p53 mutants, unlike LoVo cells (Rochette et al. 2005). Wild type p53 is generally known to interact with Smads to inhibit the transcription of numerous tumor suppressor genes. Mutant p53 has been reported to disrupt tumor suppressor TGF-β responses and reduce the transcriptional activation of major TGF-β target genes (Elston and Inman 2012). Mutant p53 has also been reported to interact with Smads but lose its DNA-binding function (Pfister and Prives 2017).

The MTT assay suggested that 40 μM Δ^9^-THC inhibited the growth of SW480 and LoVo cells, but no significant cytotoxic effect was observed at lower concentrations. Annexin V staining and morphological analysis suggested that 40 μM Δ^9^-THC treatment could induce cell death, and multiple vesicles were observed in the cytoplasm. Our previous study also showed intracellular vesicle formation and death in CBD-treated A549 human lung cancer cells, suggesting that Δ^9^-THC has anticancer effects similar to those of CBD, but the pharmacological concentration of Δ^9^-THC was estimated to be slightly higher than that of CBD (Park et al. 2022). However, FACS analysis indicated that the induction of cell death by Δ^9^-THC was not strong, indicating that physiological concentrations of Δ^9^-THC or CBD might be an important determinant in cell death or morphological changes. The expression of the overall cyclin proteins was decreased by Δ^9^-THC treatment and might be correlated with cell death and morphological changes. Δ^9^-THC induced apoptotic cell death by increasing the expression of p53, cleaved caspase 9, and cleaved PARP1. Δ^9^-THC also increased the expression of RIP1 and RIP3, which are marker proteins of pyroptosis. Given that the dead cell population induced by 40 μM Δ^9^-THC represented only a fraction of the total cell population, the anticancer effect of Δ^9^-THC might be related to a combination of mechanisms associated with cell death-related proteins. Decreases in ATP levels by Δ^9^-THC treatment were closely related to changes in mitochondrial membrane potential. In fact, the best-characterized endocannabinoids, N-arachidonoyl ethanolamine (anandamide, AEA), and 2-arachidonoylglycerol (2AG), were shown to inhibit the activity of respiratory complexes II, III, and IV and increase the activity of respiratory complex I, followed by decreases in OCR and ATP synthesis in a dose-dependent manner (Signorello et al. 2023). The endocannabinoid effect on aerobic metabolism seems to also be a CB1-mediated mechanism. Vesicle is a structure enclosed by a lipid bilayer, and also functions intercellular communication through transfer of proteins and genetic material (Bonsergent et al. 2021; Robbins and Morelli 2014). Vesicle is pivotal to signaling across synapses enabling intracellular communication in the sensory and nervous systems, suggesting an insight into how further developments could affect neurological disease treatments (Prichard et al. 2021). CBD significantly inhibited vesicle release in prostate cancer, hepatocellular carcinoma and breast adenocarcinoma, suggesting that CBD poses as a novel and safe modulator of EMV-mediated pathological events (Kosgodage et al. 2018). Our previous data suggested that CBD induced the expression of PPARγ dependent vesicle formation-related proteins, clathrin and β-adaptin (Park et al. 2022). Clathrin-coated vesicles were the most extensively characterized transport vesicles and also might be regulated by PPARγ, indicating that CBD is associated with in PPARγ-dependent vesicle formation and the induction of apoptosis. Δ^9^-THC showed ex-pression patterns similar to those of CBD, suggesting that intracytoplasmic vesicle formation is a common morphological change resulting from CBD and Δ^9^-THC treatments. We also observed that the changes due to Δ^9^-THC treatment occurred at the transcriptional level and the regulation was PPARγ dependent. The PPARγ antagonist GW9662 counteracted the antiproliferative effects induced by Δ^9^-THC, suggesting that Δ^9^-THC-induced vesicle formation may be associated with cell growth inhibition, as well as decreased metabolic capacity. Like CBD, Δ^9^-THC also has anti-tumor effects and seems to regulate several cell death proteins, as well as intracytoplasmic vesicle formation. Therefore, the individual activities of CBD and Δ^9^-THC suggest that the cannabinoids are both cytotoxic and cytostatic in nature. However, the psychotropic activity of Δ^9^-THC limits its clinical use. Recent studies suggested that Δ^9^-THC and CBD, separately or in combination, significantly inhibited the proliferation of cancer cells in a dose-dependent fashion (Milian et al. 2022). Combined treatment with both cannabinoids decreased the IC_50_, demonstrating that the combined use of Δ^9^-THC and CBD could confer greater benefits.

In summary, although the pharmacologically active concentration of Δ^9^-THC was higher than that of CBD, Δ^9^-THC regulated dual functional mechanisms, intracellular vesicle formation and cell death induction. These findings can be used to determine the best cannabinoids to improve the efficacy of cancer treatment.

### Electronic supplementary material

Below is the link to the electronic supplementary material.

Supplementary Material 1
