# Supplementary material for: Cell death induction and intracellular vesicle formation in human colorectal cancer cells treated with Δ9-Tetrahydrocannabinol
Source: Genes Genomics. 2023 Oct 14;45(12):1463–74. doi: 10.1007/s13258-023-01466-7 (PMC10682224; doi:10.1007/s13258-023-01466-7)
Supplement: Supplementary file 1 — Supplementary Material 1 [file 13258_2023_1466_MOESM1_ESM.docx]

**Supplementary Figure S1. Time dependent protein expression of untreated and vehicle control.** The Western blot images were shown in vehicle (acetonitrile) treatment. Vehicle treatment has no distinct effect on expression of cyclin proteins.

**Supplementary Figure S2. Immunostaining of clathrin expression.** Cells were treated with Δ^9^-THC and GW9662 for 24 hours and subjected to immunostaining.

Supplementary Figure S1.


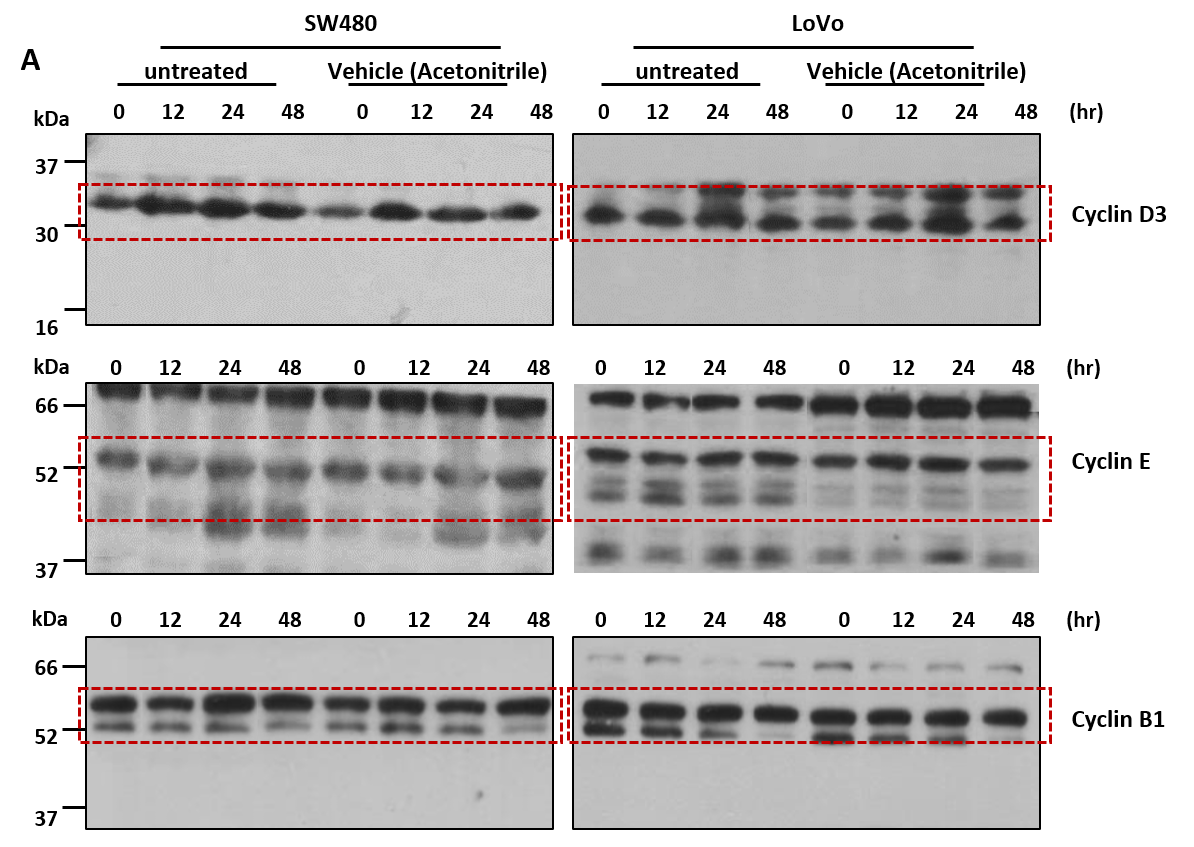


Supplementary Figure S2.


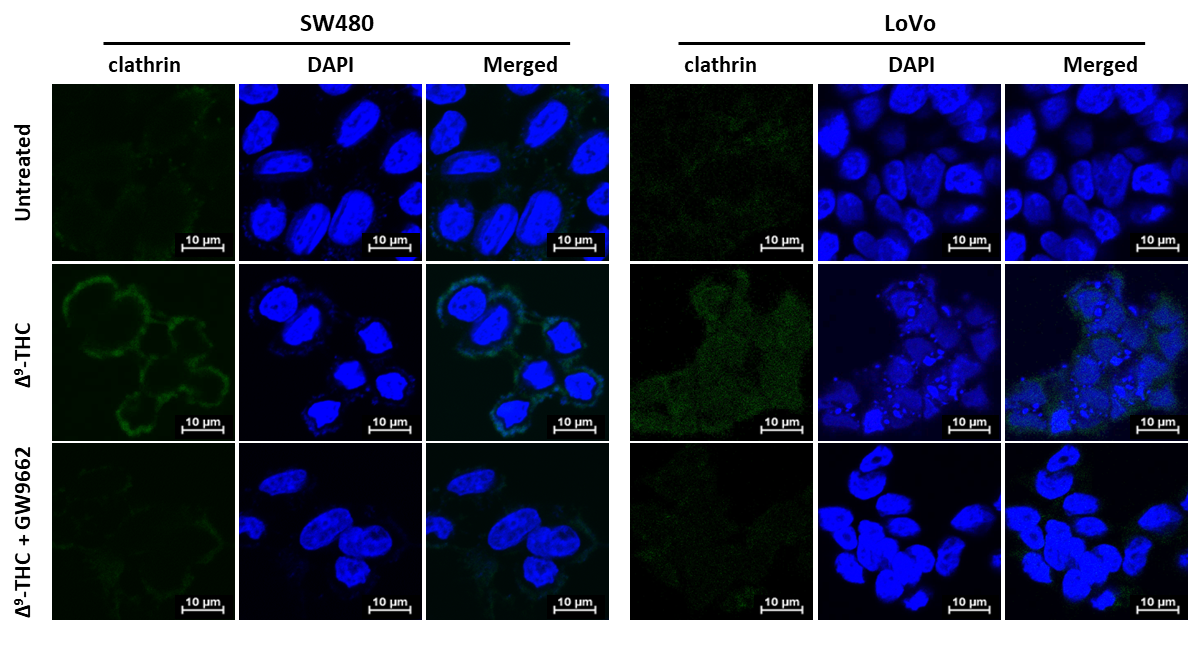


**Supplementary Table S1. Primer sequences used in qRT-PCR**

| **Gene name** | **Primer Sequence** |
| --- | --- |
| PPARG(PPARγ) | Forward : GTT CTC ATA TCC GAG GGC CA |
|  | Reverse : TGC CAA GTC GCT GTC ATC TA |
| CLTC(Clathrin) | Forward : GTC TGC CAA CAT CCG TCA GA |
|  | Reverse : AAG TGC ACA TCT GGG TCC TG |
| ACTB(β-actin) | Forward : GGA TTC CTA TGT GGG CGA CGA |
|  | Reverse : CGC TCG GTG AGG ATC TTC ATG |
